# Role of Precursor Carbides for Graphene Growth on Ni(111)

**DOI:** 10.1038/s41598-018-20777-4

**Published:** 2018-02-08

**Authors:** Raffael Rameshan, Vedran Vonk, Dirk Franz, Jakub Drnec, Simon Penner, Andreas Garhofer, Florian Mittendorfer, Andreas Stierle, Bernhard Klötzer

**Affiliations:** 1 Institute of Physical Chemistry, University of Innsbruck, Innrain 52c, A-6020 Innsbruck, Austria; 2 Department of Inorganic Chemistry, Fritz-Haber-Institute of the Max-Planck-Society, Faradayweg 4–6, D-14195 Berlin, Germany; 3 Deutsches Elektronen-Synchrotron (DESY), D-22607 Hamburg, Germany; 4 Fachbereich Physik, Universität Hamburg, D-22607 Hamburg, Germany; 5 ESRF-The European Synchrotron, Avenue des Martyrs 71, 38000 Grenoble, France; 6 Institut für Angewandte Physik, Center for Computational Materials Science, Technische Universität Wien, Wiedner Hauptstrasse 8-10, A-1040 Wien, Austria

## Abstract

Surface X-ray Diffraction was used to study the transformation of a carbon-supersaturated carbidic precursor toward a complete single layer of graphene in the temperature region below 703 K without carbon supply from the gas phase. The excess carbon beyond the 0.45  monolayers of C atoms within a single Ni_2_C layer is accompanied by sharpened reflections of the |4772| superstructure, along with ring-like diffraction features resulting from non-coincidence rotated Ni_2_C-type domains. A dynamic Ni_2_C reordering process, accompanied by slow carbon loss to subsurface regions, is proposed to increase the Ni_2_C 2D carbide long-range order via ripening toward coherent domains, and to increase the local supersaturation of near-surface dissolved carbon required for spontaneous graphene nucleation and growth. Upon transformation, the intensities of the surface carbide reflections and of specific powder-like diffraction rings vanish. The associated change of the specular X-ray reflectivity allows to quantify a single, fully surface-covering layer of graphene (2 ML C) without diffraction contributions of rotated domains. The simultaneous presence of top-fcc and bridge-top configurations of graphene explains the crystal truncation rod data of the graphene-covered surface. Structure determination of the |4772| precursor surface-carbide using density functional theory is in perfect agreement with the experimentally derived X-ray structure factors.

## Introduction

Controlled chemical vapour deposition (CVD) of well-defined, highly ordered large-area graphene layers is mandatory for a variety of potential technological applications, *e.g*. nanoscaled ultrafast field effect transistors, novel energy-storage materials, batteries, transparent conducting electrodes, etc.^1–3^. CVD of graphene has, thus, been studied extensively on a variety of metal substrates^4^. Some of these metals exhibit a rather poor solubility of carbon in the bulk, whereas others – such as the Ni(111) substrate used in this work – dissolve carbon quite well in the surface near bulk regions already at temperatures above 673 K. Poor solubility favours a surface-limited growth mechanism, proceeding via continuous decomposition of different gaseous carbon feedstocks. In contrast, substantial antisegregation of carbon into the metal near-surface and bulk regions at elevated temperatures allows for subsequent (sub)monolayer re-segregation of graphene^5^ or even multilayer re-precipitation of graphitic carbon^6^ upon cooling, depending on the experimentally provided degree of carbon supersaturation of the Ni bulk.

On Ni(111), both cases have been verified, as discussed by Dahal *et al*. in a comprehensive review on graphene-nickel interfaces^7^. The temperature-dependent growth mechanisms of graphene by chemical vapor deposition under UHV conditions by exposure to ethylene (10^−8^ to 10^−6^ mbar) are reviewed and the thermal stability of graphene and the surface-confined Ni_2_C nickel carbide are compared. At least three different growth regimes are distinguished:

(1) Below 770 K, the Ni_2_C surface carbide phase forms rapidly at the surface. The ambiguous role of Ni_2_C, serving on the one hand as a carbon reservoir for subsequent graphene growth and, on the other hand, as a kinetic obstacle suppressing the nucleation of the more carbon dense graphene^8^ is pointed out. The role of intermediate Ni_2_C on the way to graphene as a potential kinetic barrier is assigned to its reduced ability to dehydrogenate ethylene and because surface-confined Ni_2_C is a carbon-dilute (0.45 ML C) low energy line phase in the Ni–C surface-phase diagram. Therefore, it is believed to exhibit a considerable kinetic barrier of rearrangement toward graphene, since large carbon and Ni density fluctuations are required to achieve the local C-coverage of 2 ML required for graphene nucleation. As a consequence put forward in our present study, nucleation of graphene is likely to become easier once additional carbon supersaturation of the surface-near regions below the 2D Ni_2_C layer is provided.

(2) Between 770 and 920 K no stable surface carbide is formed and graphene grows on the apparently pure Ni(111) surface. In LEEM studies of the same group^9^, a carbon-denuded transition zone between the initially formed metastable Ni_2_C domains and the advancing graphene front was observed, highlighting the complex function of subsurface carbon during the transition from Ni_2_C toward the final product monolayer graphene.

(3) Between 920 and 1070 K the high mobility of C atoms in the Ni bulk renders it hard to establish a sufficient C-supersaturation of the near-surface regions, which is both mandatory for graphene nucleation and growth and for thermodynamic stabilization of already formed graphene domains. In UHV this was so far only achieved on up to 300 nm thick Ni-films, which can be more easily presaturated with carbon as compared to a bulk single crystal, at temperatures up to 1070 K^7^.

As a general trend, an increasing fraction of rotated graphene domains was observed with increasing growth temperature. Yang *et al*.^10^ suggest that, according to the Brønsted–Evans–Polanyi (BEP) relation, the activation energy for non-epitaxial graphene nucleation should be larger than for the much more stabilized epitaxial graphene, in order to explain the increasing contribution of rotated domains at higher temperatures at and above 813 K. Using LEEM, they show that perfectly epitaxial and unrotated graphene islands nucleate and grow via decomposition of the Ni_2_C surface carbide at about 673 K, which is fully in line with the results of our present study (see results and discussion section 3.). This is in contrast to a mixture of epitaxial and non-epitaxial graphene domains growing on Ni(111) with an initially partial Ni_2_C coverage, which becomes continuously replaced by graphene at 810 K. With respect to the role of near-surface dissolved carbon, they show that switching off the carbon gas phase supply does not immediately halt the ongoing graphene growth. As shown in^11^, the presence of a highly localized carbon-rich near-surface reservoir is suggested to explain the ongoing graphene formation, which proceeds in absence of the immediately decaying Ni_2_C surface carbide layer at ~810 K.

To account for the complex situation of a growth mechanism with simultaneous carbon gas phase deposition and resegregation of carbon from the bulk, a “combined” mechanism was proposed by Patera *et al*. for the temperature region below ~770 K, based on complementary *in-situ* XPS and STM measurements^12^. These authors discuss a temperature-dependent graphene growth mechanism in ethylene, switching from an in-plane single layer conversion (previously introduced by Batzill *et al*.^13^) or two layer carbide conversion below ~773 K to a direct Ni(111)-mediated conversion mechanism without intermediate carbide above 773 K. Again, at T < 773 K only epitaxial unrotated graphene directly attached to bulk Ni(111) was observed after conversion, whereas both rotated and unrotated graphene domains were observed above 773 K.

As a practical consequence of the above-mentioned salient features of temperature-dependent graphene growth on pure Ni surfaces, low growth temperatures (<773 K) are in principle desirable, mainly to avoid rotated graphene domains and to trigger optimized epitaxy. On the other hand, a lowering of the temperature will enhance the nucleation density and thus reduce the average domain size, so that a large amount of small domains with a large contributions of domain boundaries must result. DFT results reviewed in ref.^7^ suggest that at least two of the possible high-symmetry graphene binding states on Ni(111), namely the top-fcc and top-hcp Ni binding configurations, are energetically almost degenerate, so that both configurations are likely to occur and to give rise to a large contribution of domain boundaries upon completion of the full graphene layer (see also results and discussion section 3.). In order to reduce the nucleation density, to modulate the kinetic barrier between Ni_2_C and graphene, and to modulate the solubility/supersaturation of carbon in the near-surface regions, Weatherup *et al*. successfully introduced Au doping of Ni surfaces to achieve deliberate kinetic control of graphene growth with respect to thickness, attainable coverage, optimized epitaxy, domain size, domain orientation etc.^14^.

The other extreme of the “carbon supersaturation scale” of Ni(111) is represented by a study of Jacobson *et al*.^15^, which was achieved by C-supersaturation with the highly carburizing CVD agent toluene at ~923 K. Upon cooling of this highly carbon-enriched state of the near-surface regions, a large contribution of metastable structures, the most prominent being a layered Ni(111)-Ni_2_C-rotated graphene structure, was observed, likely because of fast and rich carbon segregation. The given interpretation of the role of the Ni_2_C surface carbide as a source of graphene grain rotation was later questioned, and for the cooling process a post-segregation of “interlayer” Ni_2_C domains beneath already rotated grown graphene domains on Ni(111) - which exhibit intrinsically weaker net binding to the substrate as compared to the epitaxial domains - was proposed as an alternative explanation^7,12^.

Despite the fact that on Ni(111) no direct evidence for 3D-crystalline carbidic graphene precursors such as Ni_3_C or other carbidic Ni-C phases has been deduced so far - *e.g*. from diffraction experiments, which also holds for our present study - the above-reviewed Ni(111) surface science studies and also our present work suggest the formation of a more or less carbon-rich “quasi-3D” Ni-C subsurface reservoir as a necessary precondition for graphene growth. In this context, an amorphous carbidic Ni_x_C_y_ precursor in between the energetically most stable hexagonal Ni_3_C bulk carbide polymorph and graphene has been postulated recently on the basis of quantum chemical molecular dynamics (QM/MD) simulations of graphene formation^11,16^, which would be necessarily invisible in diffraction measurements. Nevertheless, the critical role of local carbon supersaturation within this amorphous precursor for the ongoing graphene nucleation and growth was highlighted.

The central aim of this study is, thus, to revisit and clarify the issue of a coexistence of near-surface dissolved and carbidic Ni-C species in a sufficiently C-supersaturated scenario to induce graphene growth. Our approach using *in situ* surface X-ray diffraction provides a look at both the supersaturated carbidic precursor and the resulting graphene-covered Ni(111) surfaces at long-range ordering, thus complementing the local microscopic measurements of the Ni_2_C carbide and graphene structures presented in previous STM works. Moreover, we aimed at an unambiguous experimental clarification of the suspected Ni(111)-Ni_2_C coincidence cells denoted either as |2772| or |4772|, which is still lacking from the so far known details of LEED and STM-FFT data (see ref.^17^ and literature therein). Also, subtle, but potentially graphene-growth-relevant, changes of the Ni_2_C carbide superstructure with increasing ethylene exposure at T~673 K, being necessarily accompanied by increasing supersaturation of the Ni(111) near-surface regions with dissolved C, were anticipated. Moreover, the SXRD data of intermediate stages between the supersaturated carbidic precursor and the final graphene-covered Ni(111) surface were expected to reveal if rotated graphene domains and/or layered Ni_2_C-(un)rotated graphene regions are measurably abundant below 773 K. Regarding the choice of the ethylene CVD temperature, we aimed to revisit the complex structural scenario just “at the edge of graphene growth” at and above 673 K in the 10^−6^ mbar pressure range, as chosen previously by Patera *et al*.^12^. Under these conditions, a complete Ni_2_C layer is still stabilized in presence of the ethylene gas phase, but it can already convert quasi “by chance” (i.e. via stochastic graphene nucleation) to a full monolayer of epitaxial graphene, as postulated in ref.^7^.

## Methods

### Sample preparation and Surface X-ray Diffraction

The surface X-ray diffraction experiments were performed at beamline ID03 of the European Synchrotron Radiation Facility (ESRF), France^18^ using an X-ray energy of 18.0 keV. A polished single Ni(111) crystal was mounted in a dedicated UHV setup, which is connected to a 6-circle diffractometer. This sample chamber enables cleaning of the surface by sputter-anneal cycles, involving heating in 5 × 10^−7^ mbar O_2_ and final desorption of oxygen, exposure to ethylene and analysis of the surface composition by Auger Electron Spectroscopy (AES). The temperature was measured with a thermocouple directly spot-welded to the side wall of the disk-shaped sample, which leads to an estimated off-set of at maximum 10 K. The gas pressure was measured with a state-of-the art calibrated ionization gauge using the gas-specific sensitivity factors provided by the manufacturer. According to the specifications, the error at a pressure of 1*10^−6^ mbar is around 5%.

X-ray scattering from surfaces gives rise to crystal truncation rods (CTRs) and surface rods (SRs), where the former include a contribution from the bulk and the latter arise solely from the topmost atomic layer, which can have different periodicity and symmetry than the bulk underneath. Integrated diffraction intensities were obtained in the so-called stationary mode and converted to structure factors F in a standard way^19^. Structural models were refined against the experimental structure factors using the program ROD^20^.

Several sample preparation conditions were chosen, resulting in quasi-clean, purely Ni_2_C surface-carbide covered, strongly C-supersaturated Ni_2_C covered, and eventually graphene-covered surfaces, namely: 7.5 L ethylene deposited on the quasi-clean Ni(111) crystal at 573 K, resulting in a single layer Ni_2_C superstructure with ~0.45 ML carbon coverage, 379 L ethylene at 673 K, 1353 L ethylene at 723 K, and eventually 6316 L ethylene, first at 773 K for 1 h (corresponding to 5402 L) and then at 673 K for 10 minutes in 2 × 10^−6^ mbar ethylene (further 914 L). The latter preparation yielded the metastable “carbon-supersaturated carbidic precursor state” and spontaneously converted to a full (2 ML carbon) graphene coverage by slight thermal annealing to 703 K.

We use the term “quasi-clean” here to emphasize the fact that it is practically impossible to obtain truly clean Ni surfaces, because the bulk of the material contains a virtually infinite amount of carbon which can segregate to the surface. The quasi-clean state reported here refers to a Ni(111) surface prepared in such a way that the carbon content is considerably below the saturation level to start forming graphene or Ni_2_C.

### DFT Calculations

The calculations were performed using the VASP code^21,22^, using PAW potentials^23^, the PBE exchange-correlation functional^24^ and a cutoff energy of 400 eV. The surface carbides were modelled by a single carbide layer supported on three Ni layers. The structures were relaxed until the residual forces were smaller than 0.02 eV/A, with the two lowest Ni layers fixed during the relaxation. A 2 × 2 × 1 k-point mesh was employed for the integration of the Brillouin zone. For the calculation of the surface energies, Ni atoms are referenced to bulk Ni and C to graphite, respectively.

## Results and Discussion

### Quasi-clean Ni(111) surface

**Figure 1:**
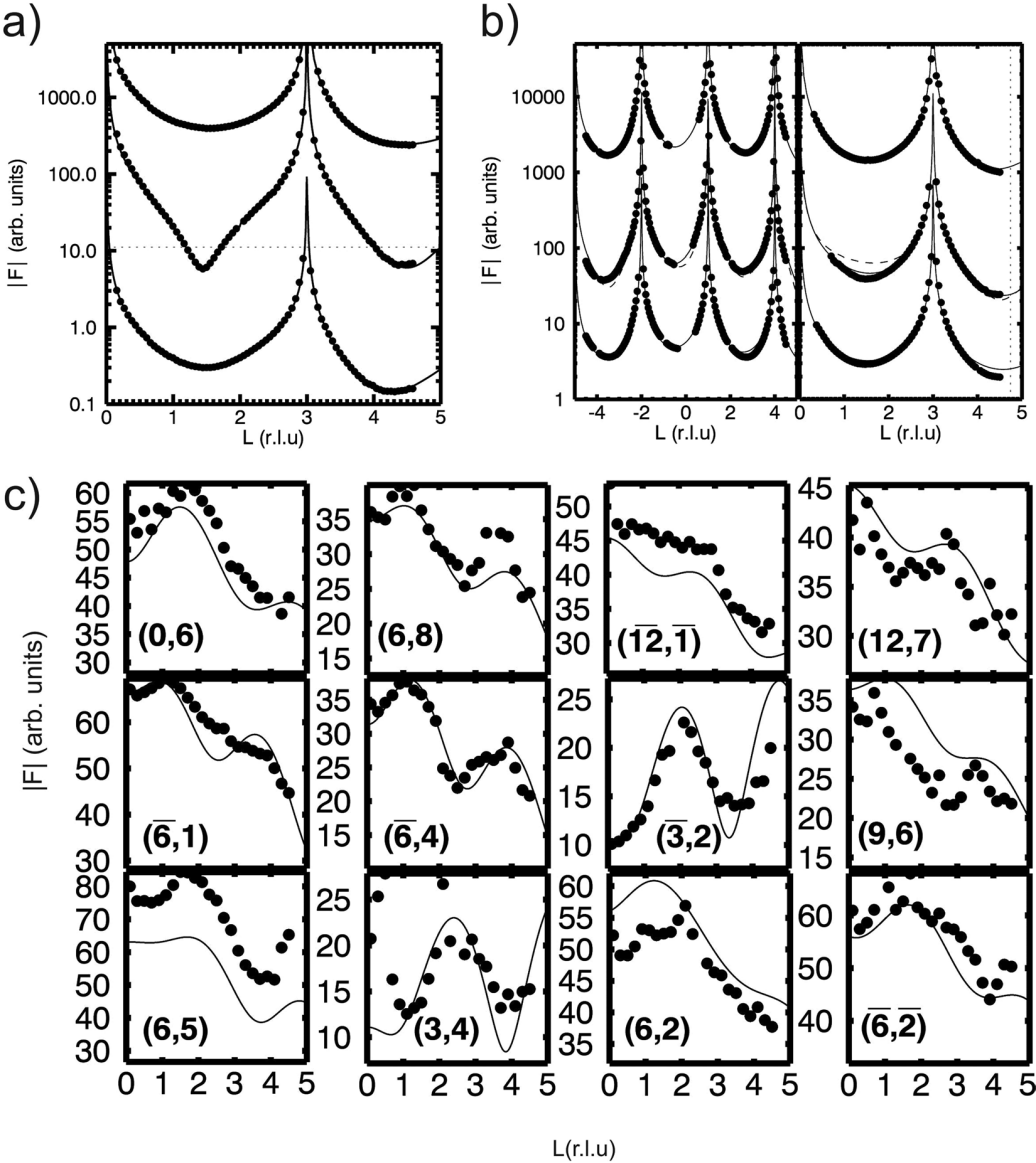
Crystal truncation rod data and fits of different sample preparations. (**a**) Specular (0, 0) rod data of the quasi-clean (top), graphene (middle) and carbide (bottom) prepared Ni(111) surface. (**b**) Selected CTR data and fits. The left column shows (1, 0) and the right (1, 1). Top to bottom are shown: quasi-clean, graphene and carbide. The solid lines show the best fits as discussed in the main text. The simulation for a single ‘top-fcc’ graphene domain is also indicated (dashed line). (**c**) Carbide rods and fits using the structure model obtained from DFT as explained in the text.

**Figure 2:**
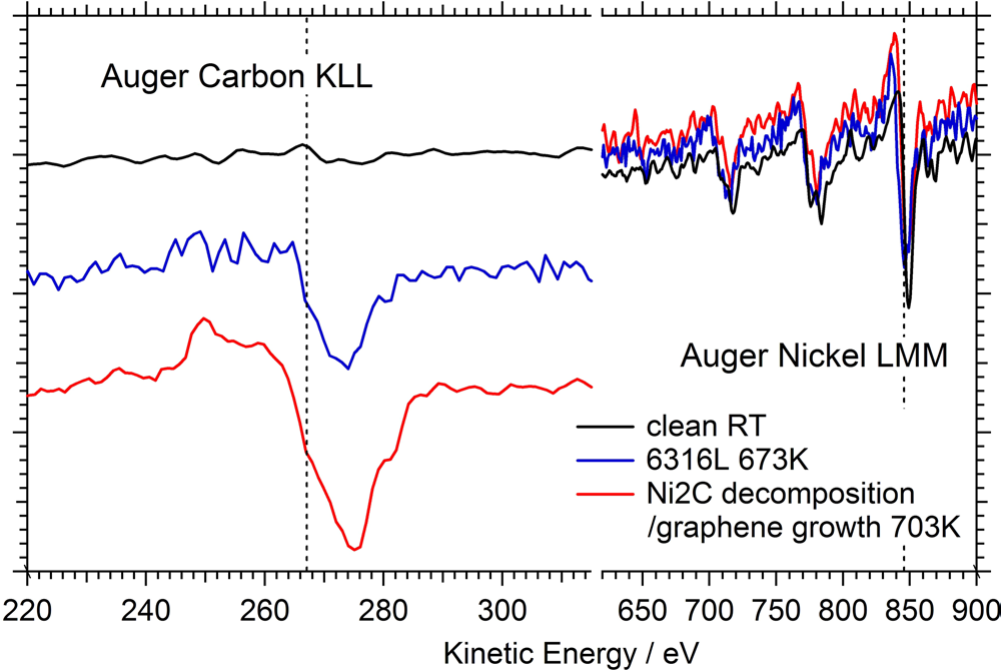
Auger electron spectra of the carbon KLL-transition (left) normalized to the respective nickel LMM intensity (right). Black line: clean sample; blue line: metastable supersaturated precursor; red line: graphene-covered surface after precursor decomposition at 703 K in vacuum.

In order to compare the diffraction and Auger Electron Spectroscopy (AES) data between different preparations, we also include the results concerning the quasi-clean surface. Figure 1 shows the experimental data and best fit results of quasi-clean Ni(111). Figure 2 shows the corresponding AES data. The crystallographic structure model consists of a 111-oriented fcc surface unit cell. Several structural parameters concerning the surface Ni atoms were refined and are listed in the Supplementary Information, Table S1. Most notably, a very small outward relaxation of the top most Ni is found. The best fit was obtained when refining the occupancy of the topmost Ni layer, resulting in a value of 0.95(1). The fact that this value deviates from 1, as would be expected for a perfect surface, is an indication of either surface roughness or the presence of residual carbon. The technique of SXRD is sensitive to atomic surface roughness^25^, which can be modelled by ‘missing’ electron density and the value obtained for the occupancy here would correspond to a root-mean-square (rms) roughness less than 0.5 Å, *i.e*. to a very smooth surface. If the refined value for the occupancy would be interpreted as resulting from residual carbon, one can calculate the carbon concentration from the difference in atomic scattering factors between Ni (Z = 28) and C (z = 6) to be about 6%. The AES spectrum of the quasi-clean state (see Fig. 1) does not show a clear C signal, which means that its concentration is below the detection limit of roughly 5%. The results of the quasi-clean state therefore indicate that the surface is very smooth and possibly still contains a few percent of carbon.

### Surface Carbide

**Figure 3:**
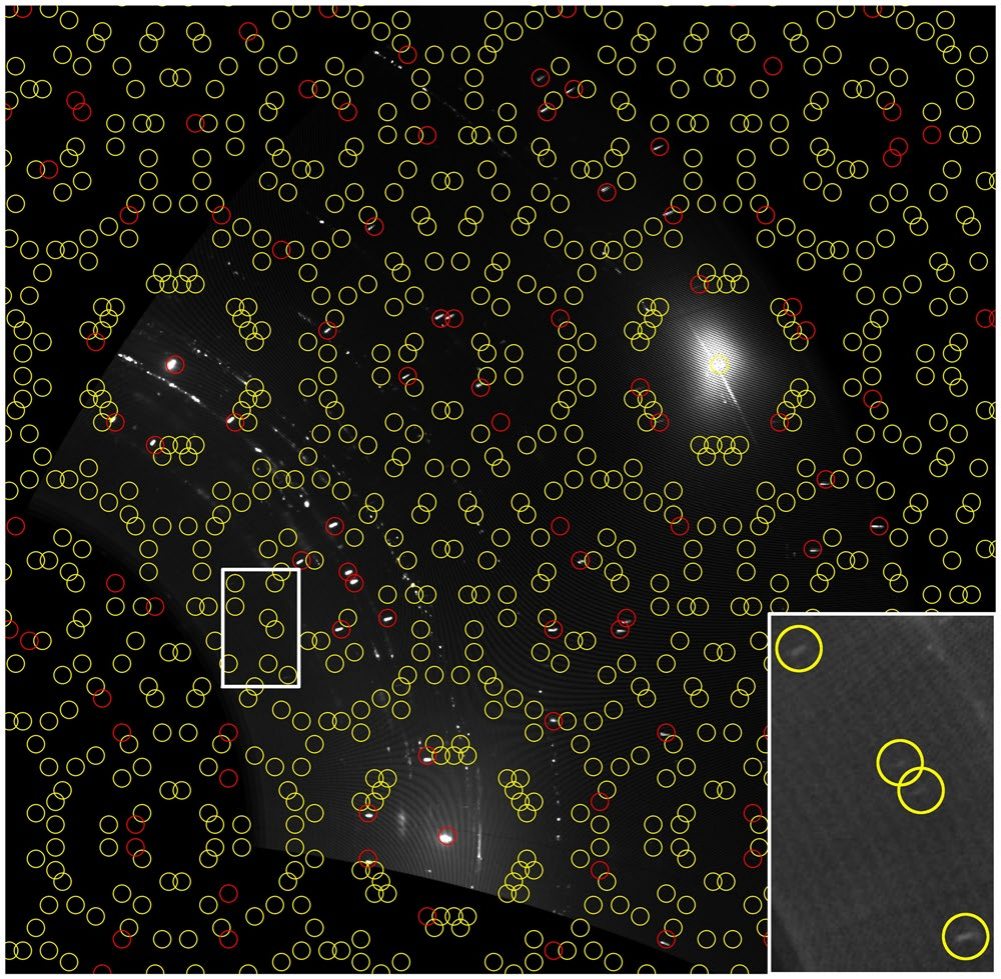
In plane (hk) X-ray diffraction map of the supersaturated surface carbide precursor on Ni(111) after 6316 L ethylene at 773/673 K. The red circles indicate the expected position of the main |4772| coincidence cell reflections and its 6 symmetry related domains, along with the |4772|-specific peak splitting, which is not visible in the low-coverage surface carbide preparations (*e.g*. 7.5 L ethylene deposited on the quasi-clean Ni(111) crystal at 573 K). The yellow circles mark the positions of the expected additional weak spots appearing due to the large |4772| coincidence cell. The three brightest spots in the figure stem from the underlying Ni(111) substrate (1, 0), (0, 1) and (1, 1) CTRs. The inset proves the existence of the weak spots originating from the large |4772| coincidence cell.

Both the usual surface science preparation of the surface carbide (7.5 L) and the 6316 L supersaturation experiment yield a typical Ni_2_C surface carbide diffraction pattern, which is relatively complex and composed of many spots. In literature, several unit cells and domain structures, based on coincidence lattices with the Ni(111), have been proposed. The dominant structural motif consists of a nearly square ~0.5 nm^2^ mesh, which is built up from 2 carbon atoms, arranged in a centered way at the corners and the middle of the unit cell, whereby the central carbon atom is surrounded by 4 Ni atoms. The Ni atoms have the freedom to rotate around their central C atom, thereby making up a so-called clock-reconstructed network. In fact, as early as 1969^26^, it was found by LEED that the actual carbide superstructure unit cell is larger than the dominant nearly-square top layer lattice, due to the occurrence of weak spots close to the main reflections and spots at relatively low scattering angles near the direct beam. At that time, a |2772| coincidence cell was proposed and later the larger |4772| superstructure cell was postulated^17^. Because of the P3m1 symmetry of the underlying Ni(111) surface, such a larger cell of lower symmetry fits in different ways on the substrate. Due to the 6 symmetry operations valid in this planar space group^27^, 6 co-existing carbide domains appear on the Ni(111) surface. The diffraction pattern shown in Fig. 3 can be only explained by a prevalence of the |4772| coincidence cell, which displays weak but detectable spots besides the main reflections and appears 6-fold due to the domain structure. The strong main reflections (red circles in Fig. 3) are related to the nearly square Ni_2_C structural motif and the weaker spots (yellow circles in Fig. 3) due to its modulation within the larger |4772| cell. In previous LEED studies, resolution limitations hindered the exact determination of the coincidence cell. Due to the small difference in lattice parameters between the two coincidence lattices, i.e. $\sqrt{39}\times \sqrt{39}$ (|2772|) and $\sqrt{39}\times \sqrt{37}$ (|4772|), for the |2772| some peaks from different domains overlap and results in a typical 5-fold splitting of the main peaks. In particular, the splitting of the surface carbide peak along the (1, 1) direction of the Ni substrate is a hitherto unobserved signal, which is a direct evidence that the |4772| cell is the dominant one, at least after the 6316 L ethylene exposure at 773/673 K. No traces of the |2772| cell, which would exhibit no such spot splitting, are detectable within our error limit (at maximum 5% of the full carbide coverage assuming equally well-ordered |2772| domains). Nevertheless, as will be shown later in section 5, the peak splitting appears incomplete or less pronounced after smaller ethylene exposures. In these cases a coexistence of |4772| and |2772| domains in similar quantities cannot be excluded.

Figure 3 shows a magnified section of the hk-map to better visualize this peak-splitting feature. It has to be noted that the observation of this fingerprint-like spot splitting depends on the sample preparation conditions, as discussed in more detail further on; the splitting may sometimes appear much less pronounced resulting in an apparently single, broadened, peak. Nevertheless, our high resolution X-ray study now clearly identifies the |4772| cell as the correct and best-ordered one, at least after extended exposures at elevated temperatures around 673 K.

This trend is confirmed by the DFT calculations, which also predict a slightly higher stability of about 6 meV/Å^2^ of the $\sqrt{39}\times \sqrt{37}$ (|4772|) surface compared to the $\sqrt{39}\times \sqrt{39}$ (|2772|) surface carbide at the chemical potential of graphite. Hence, while both surface carbides show a similar structural motiv, the slightly higher C content of the |2772| is compensated by a less favorable binding geometry: While the calculations predict a similar average Ni-C distance of 1.88 Å for the |4772| and 1.83 Å for the |2772|) *within* the carbide layer, the |4772| yields a smaller mismatch to the metallic Ni layer below, thus facilitating the formation of additional bonds. In addition, we find a strong buckling of both the Ni and the C atoms in the carbide layer: In the case of the |4772| surface the Ni atoms in the Ni_2_C layer are buckled for 0.16 Å, slightly smaller than the value of 0.20 Å for the the |2772| surface. The buckling is more pronounced for the C atoms, with values of 0.65 Å (|4772|) and 0.99 Å (|2772|). The binding of the surface carbide to the metallic Ni layer below also induces a buckling in this layer, with a comparable value of 0.29 and 0.32 Å for the |4772| and |2772|) interface, respectively.

**Figure 4:**
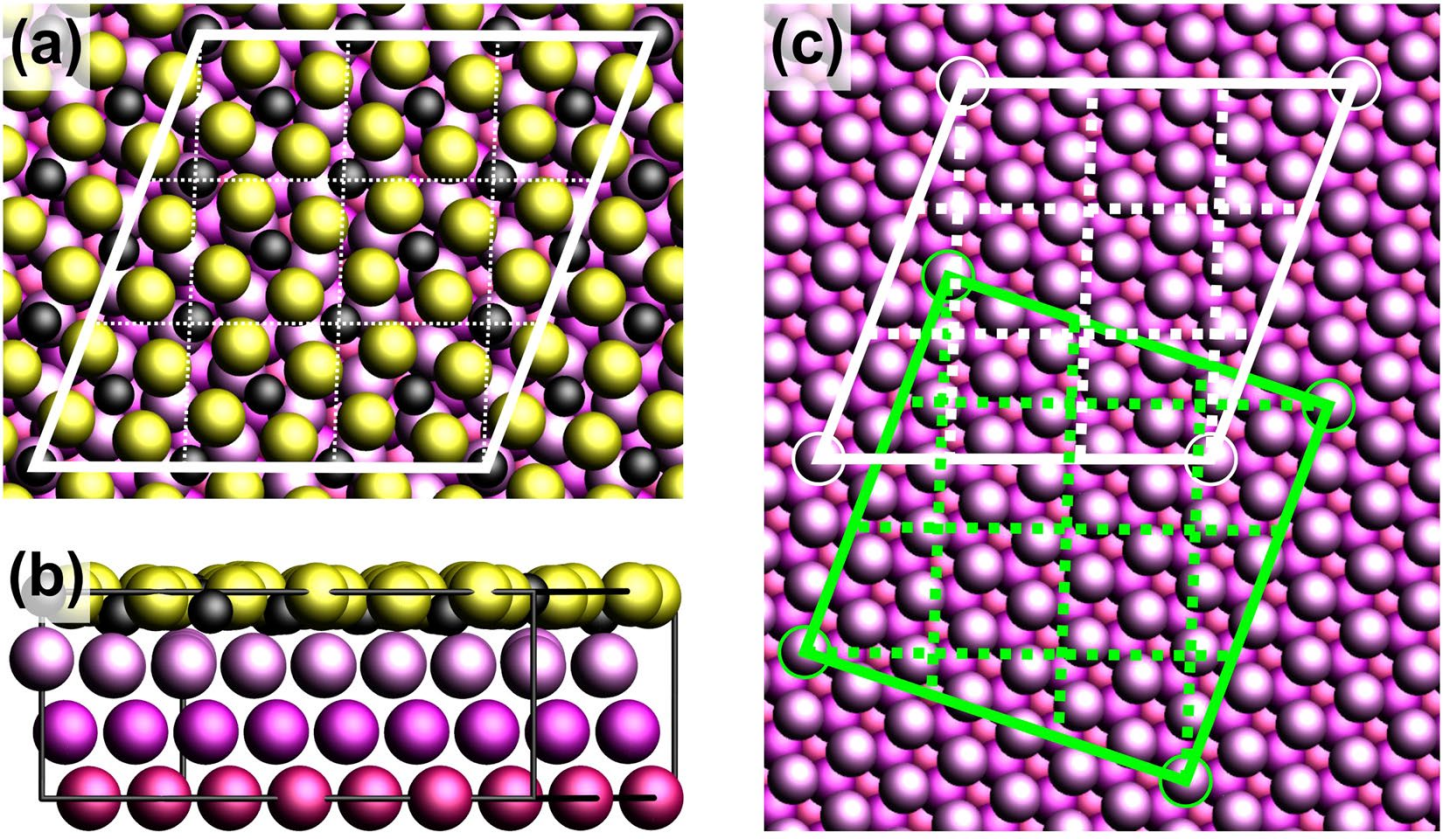
Ball model of the atomic structure of the |4772| carbide phase as obtained by DFT and further refined by SXRD. (**a**) Top view, showing substrate Ni (magenta, with a gradient indicating the depth from the surface), carbide Ni (yellow) and C (black). The |4772| coincidence cell is indicated (thick white) as well as the nearly-square dominant structural motif (dashed white). (**b**) Side view along the (010) direction showing the 2D nature of the carbide phase and the corrugation of the Ni and C atoms. The carbon atoms (black) lie closer to the underlying nickel layer than the Ni atoms which belong to the surface carbide (yellow). The 2nd Ni layer from the surface contains atoms which basically reside at bulk lattice positions. The layer just below the carbide contains Ni atoms of which the positions are slightly different from those in the bulk, resulting in the |4772| supercell. (**c**) Ni(111) surface and the indication of the coincidence cells |4772| (white) and |2772| (green). The underlying nearly-square mesh is indicated (white dashed) and is seen to be nearly identical for both larger coincidence cells.

In a next step, the CTRs and surface rods (SRs) are analyzed and compared with the DFT model of the |4772| cell, see Fig. 1. For the fitting of the Ni_2_C reflections, data from two preparations of the Ni_2_C top layer were combined: 7.5 L at 573 K and the supersaturated precursor (6316 L at 773/673 K). This assured a data set large enough to fit all the features of the 2D top layer structure simultaneously and with strongly enhanced accuracy. The diffraction indices (h,k) are related to the |4772| unit cell. As a starting point, we have used the results from the DFT calculations and refined this model further. The respective model is shown in Fig. 4. The modulations along L on the surface carbide rods apparently stem from a structure which is not described by a single monolayer on top of an otherwise unaltered bulk Ni(111) layer. Interestingly, the DFT results, obtained from the carbide monolayer model, describe these modulations very well and indicate that they are the result of slight in-plane relaxations of the Ni layer underneath the surface carbide. Such in-plane relaxations break the typical Ni(111) planar symmetry and result in small, but non-negligible, Fourier components at the otherwise non-existing |4772| lattice points. These results nevertheless indicate that the surface carbide top layer is truly 2-dimensional, i.e. never more than one monolayer thick.

Special care needs to be taken concerning the symmetry of the domain structure when using both the CTR and SR data at once in the refinement. We have measured SRs from one domain only. At the same time, different domains contribute to the CTRs. The refinement was performed by assuming an equal coverage for each of the domains and by transformations based on the symmetry of the Ni(111) surface as described above. A few fit parameters have been further refined, such as the distance of the whole Ni_2_C slab to the substrate and the rumpling between the C and Ni layer as well as the rotation angle of the clock-reconstructed motif. The average distance between the carbon and nickel atoms in the Ni_2_C layer is about the same as obtained from DFT and the average rumpling appears to be 0.1(4) Å smaller. Due to the relatively large error and the enhanced Debye-Waller parameters of around 0.4 Å^2^, it is concluded that the differences between the DFT and SXRD results are hardly significant and that the proposed structural model has excellent agreement. Table S2 in the Supplementary Information summarizes the fractional co-ordinates of the refined Ni2C |4772| coincidence cell, along with the refined Debye-Waller parameters.

### Graphene

**Figure 5:**
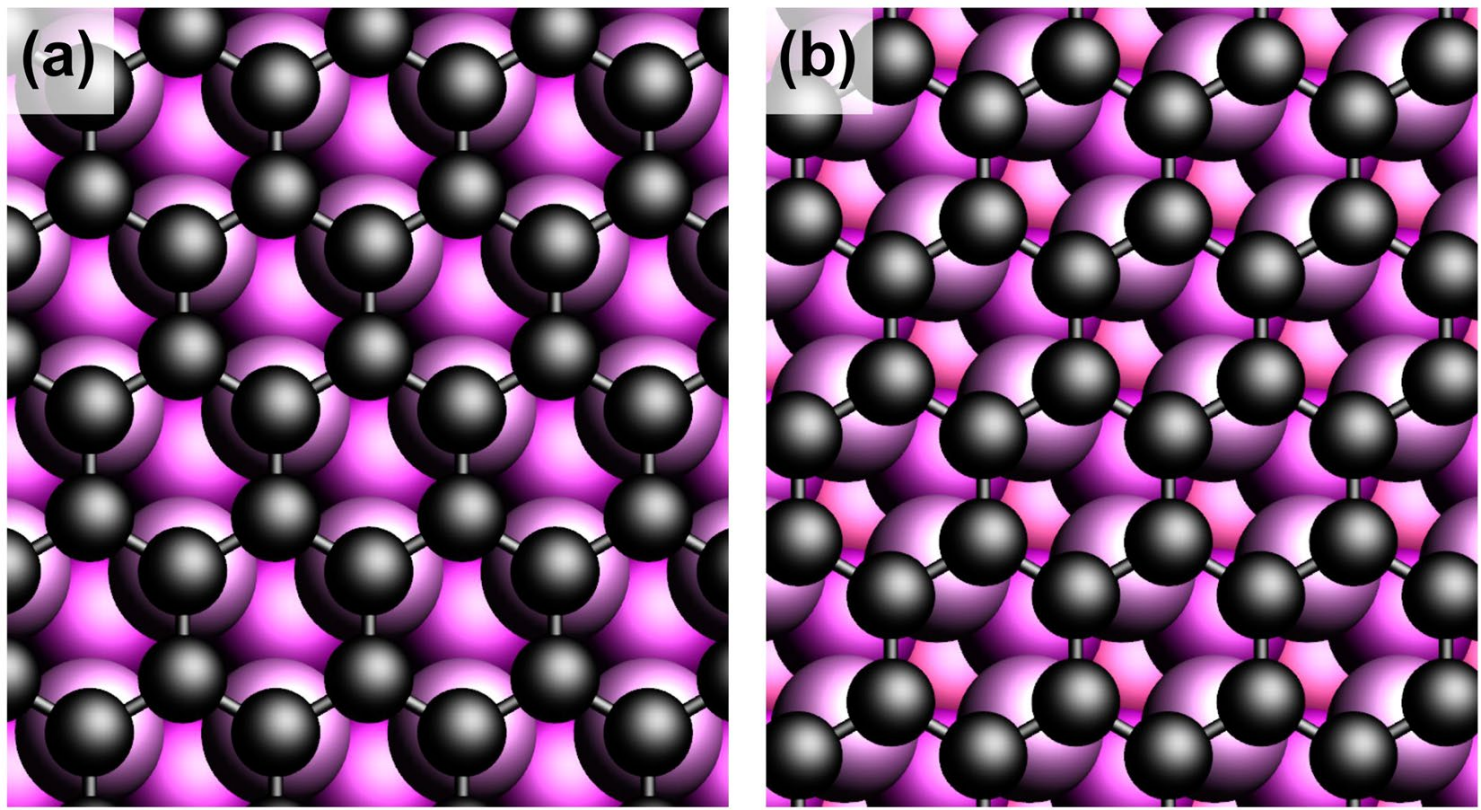
Schematic representation of the two fitting-relevant commensurate stacking geometries of graphene on Ni(111), namely (**a**) the top-fcc and **(b**) bridge-top configurations. The best fit with the SXRD data was obtained by assuming a 50:50 domain co-existence.

Here, we describe the structure of the fully surface-covering graphene layer obtained after thermal annealing of the metastable supersaturated precursor state at 703 K. Due to the near-perfect lattice match between graphene (a = 0.2456 nm) and the Ni(111) surface (a = 0.2489 nm), the former grows in a perfectly epitaxial fashion on the substrate. Because graphene has two atoms in the unit cell and Ni(111) has one atom per surface cell, there exist in principle six different atomic stacking possibilities. Previously, two of these stacking possibilities have been identified to co-exist for energetic stability reasons^28^, the so-called top-fcc and bridge-top configurations, as shown in Fig. 5. The most pronounced signature for the presence of graphene is seen in the shape of the specular rod, see Fig. 1. The distinct shape around the minimum at L = 1.4 is perfectly described by a model in which the Ni(111) surface is fully covered by a (2 ML carbon) graphene layer. The specular rod is particularly sensitive to the distance of the graphene to the substrate, which is determined to be 0.2088(5) nm. This value corresponds very well with the almost equal distances obtained for top-fcc and top-bridge stackings, as derived from DFT^28^. The other four stacking possibilities would result in much larger distances, which disagree with the value obtained by SXRD. Due to the perfect epitaxy of the domains with the Ni(111) surface, the graphene scattering contributes exactly to all the CTRs of the substrate; reciprocal space maps did not reveal any signs of abundant randomly rotated graphene domains, which would show up as powderlike diffraction rings, nor of any graphene surface rods. Interestingly, the interference between the scattering contributions of the two co-existing domains leads to almost complete extinction for diffraction with non-zero in-plane momentum transfer, i.e. for all rods except the specular. By calculating the structure factor of the graphene scattering, under the assumptions that the two domains both cover 50% of the surface and that the height above the Ni surface of all the 4 carbon atoms is identical, one obtains: ${F}_{G}(10L)=\frac{1}{2}{f}_{C}{e}^{i2\pi {z}_{G}L}{e}^{i\frac{2}{3}\pi }$ and ${F}_{G}(11L)\,=\,0$, with L the diffraction index along the rods, *z*_*G*_ the height of the graphene layer above the substrate, *f*_*C*_ the atomic scattering factor of carbon. The total structure factor, which is used to fit to the experimental data, is calculated as: ${F}_{{tot}}({hkL})=|{F}_{{bulk}}({hkL})+{F}_{G}({hkL})|$, with *F*_*bulk*_(*hkL*) representing the CTR of the Ni(111) surface. The mutual destructive interference of the graphene domains results in a very small contribution to the (1, 0) rod and even no contribution to the (1, 1) rod. In contrast, the scattering contribution to the (0, 0) specular rod can be as high as 2*f*_*C*_, which occurs in the minimum at L = 1.4 along the specular rod, resulting in nearly complete destructive interference. Figure 1 also shows a simulation with a model in which only the top-fcc domain is taken into account and, clearly, the fit becomes worse. The in- and out-of-plane carbon atom Debye-Waller parameters ${B}_{\Vert }$ and ${{B}}_{\perp }$, respectively, which represent a measure of the static disorder, show enhanced values. For the out-of-plane direction this is an indication of slight rumpling, most likely induced by the different carbon adsorption sites in the two domains. Possibly, a related domain boundary ridge structure such as that proposed to separate the fcc and hcp domains of graphene on Ni(111)^29^ might be a candidate to explain this observation. For the in-plane direction it is probably related to the domain wall density, but because the scattering contribution is already very low, refinement of ${B}_{\Vert }$ is not very reliable and is not very useful for further quantification of the domain structure. The refined occupancy parameter of the last Ni layer results in a value of 0.96(1), which indicates - besides the aforementioned roughness contribution - a residual surface-near concentration of dissolved carbon in the range of a few percent even after the growth of the full graphene layer. Table S3 in the Supplementary Information summarizes the fractional co-ordinates of the refined graphene-covered Ni(111) surface, along with the refined Debye-Waller and occupancy parameters.

### Metastable supersaturated carbidic/dissolved carbon precursor state: formation, stability and conversion to graphene

**Figure 6:**
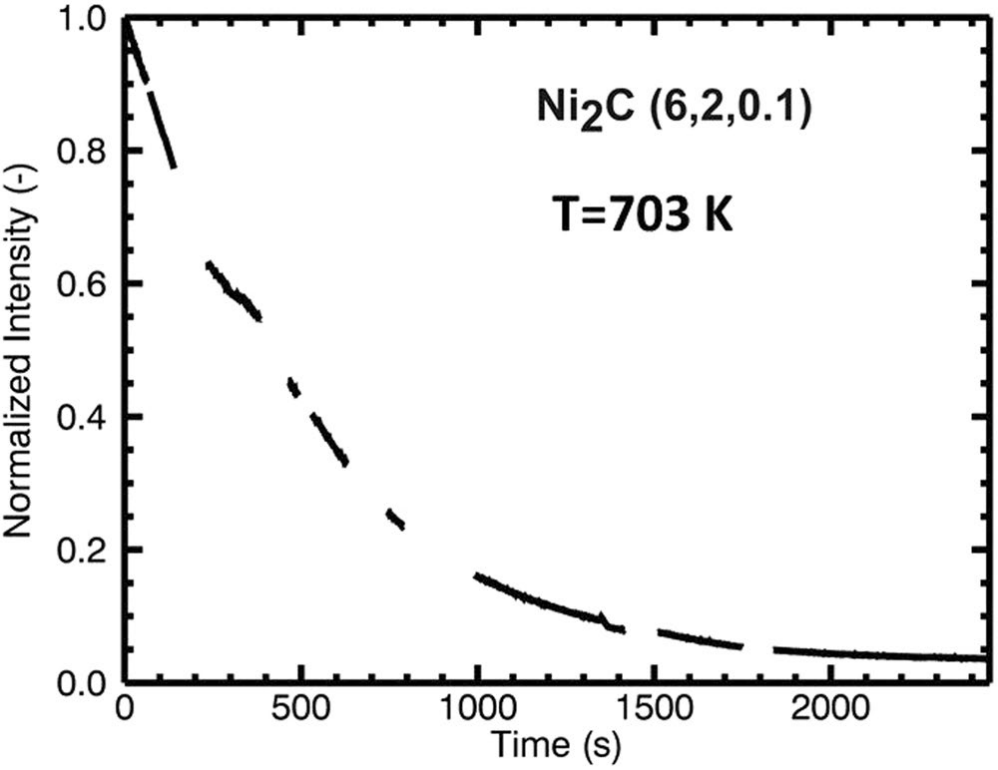
Time evolution of the surface carbide (6, 2, 0.1) reflection during thermal annealing (703 K) of the metastable supersaturated carbidic precursor (grown in 6316 L ethylene at 773/673 K) toward graphene on the Ni(111) surface. The normalized intensity decrease indicates that the ordered Ni_2_C surface phase disappears completely. The gaps in the measurement arise from the fact that the sample alignment was checked and optimized several times.

The aim of this experiment was to characterize the Ni(111) surface in its fully carbon-saturated state prior to the nucleation/growth of graphene, and, in due course, to follow the temperature-induced transformation of the deeply “carbidized” surface toward graphene in the absence of external gas phase supply of carbon (i.e. under good UHV conditions). For this purpose, the sample was exposed for 1 h at 773 K to 2 × 10^−6^ mbar ethylene (5400 L) to obtain “deep” saturation with bulk/ near surface dissolved carbon. After decreasing the temperature to 673 K, additional ~10 minutes of exposure to ethylene followed, resulting in a total exposure of 6316 L and inducing a fully surface-covering Ni_2_C layer. The Auger spectrum after this preparation and pumping off is shown in Fig. 2. The C-signal exhibits the typical carbide fingerprint^30^. At the same time, the SXRD pattern (see Fig. 3) shows the presence of the |4772| carbide reflections. Then, the sample was quickly heated to and held at 703 K in UHV. During this isothermal heating period, the intensity of a single surface carbide reflection (6, 2, 0.1) was monitored and is shown in Fig. 6.

After heating for about 2000 s, the Ni_2_C intensity disappeared completely. The corresponding AES spectrum, shown in Fig. 2, shows that the carbon signal switched to the typical fingerprint of graphene/graphite and gained in intensity, due to surface segregation of dissolved carbon toward the graphene top layer. Indeed, further SXRD measurements confirmed that graphene had formed, as seen in the typical shape of the specular CTR (shown in Fig. 1, and results and discussion section 3.).

This result is interpreted in the context of two previous studies: (1) 673 K ethylene-exposed surfaces can be converted quasi “by chance” (i.e. via stochastic graphene nucleation) to a full monolayer of epitaxial graphene, as discussed in ref.^7^. By slight heating to 703 K under UHV, this “stochastic” process was deliberately induced, as the gas phase stabilization of the top Ni_2_C layer is switched off and the supersaturated carbidic precursor is at the same time thermally destabilized. (2) In a LEEM study of the same group^9^, a carbon-denuded transition zone between the initially formed metastable Ni_2_C domains and the advancing graphene front was observed at 823 K, but not at 773 K. This leads to a characteristic time-dependence of the Ni_2_C decay and the subsequent graphene growth process. At around 873 K, immediate decay of the surface carbide is followed by a delayed growth process of the graphene layer (within minutes). In contrast, at 773 K the time-divergence of these processes is much smaller, and an apparently simultaneous carbide-decay/graphene growth process is observed, corresponding rather to the „interfacial“ carbide-graphene conversion/growth picture drawn in the work of Patera *et al*.^12^. Our measurement does not allow to resolve the time evolution of the graphene top layer, but the analysis of the specular (0, 0) rod directly after the 2500 s thermal annealing experiment of Fig. 6 showed an already complete 2 ML carbon coverage, i.e. a fully surface-covering graphene layer at the surface. Thus, we observe a comparably small time delay between carbide decay and graphene growth, rather as reported for graphene growth at 773 K in^9^. The conclusion is that our 703 K annealing experiment is still dominated by, or at least not far off the „direct conversion mechanism“ put forward in^12^.

**Figure 7:**
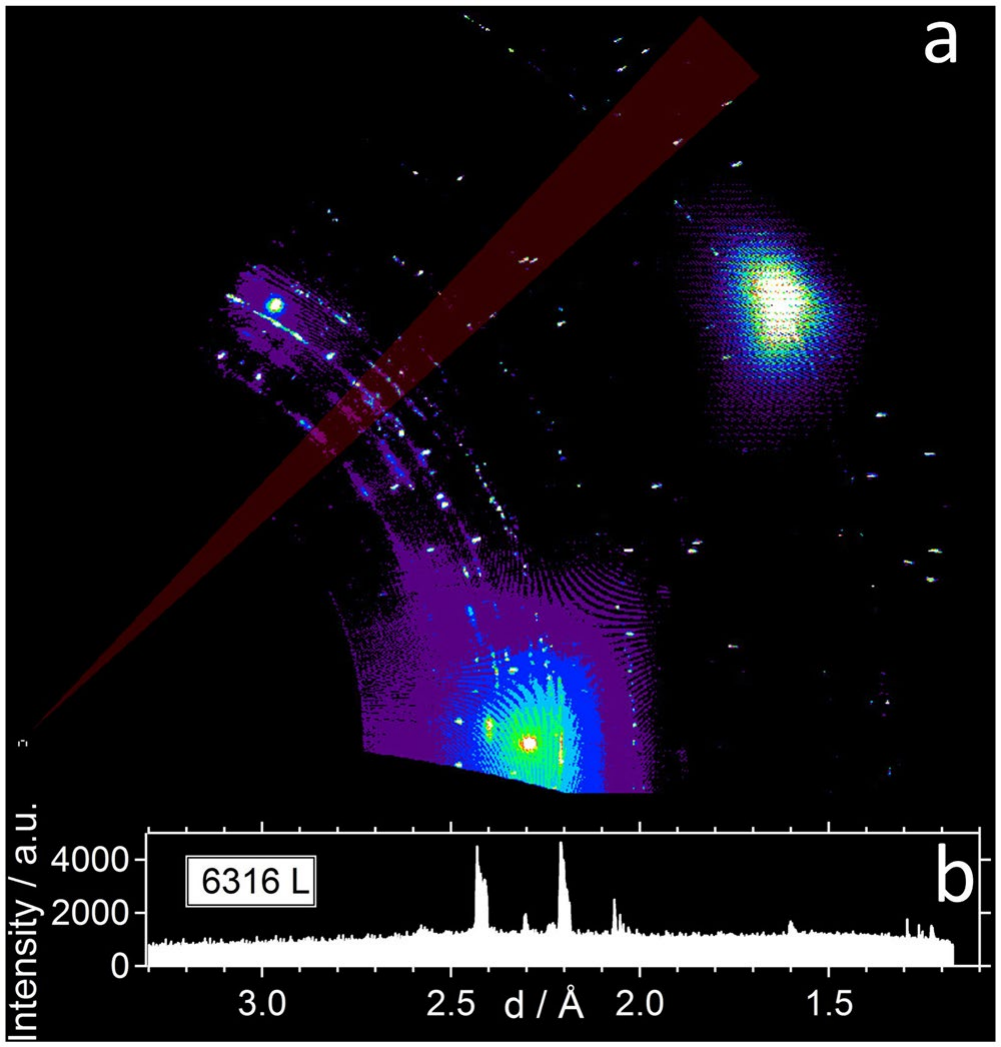
(**a**) hk – map of the supersaturated carbidic/dissolved carbon precursor exhibiting ring-like diffraction features. (**b**) highlighted diffraction ring-like intensities with a minimum Ni_2_C/Ni(111) spot intensity contribution, represented as a “powder diffractogram”. The integrated detector raw intensity data within the area marked by the red transparent bar were used to plot panel (**b**). The optically enhanced b/w contrast in panel (**a**) provides no scalable measure of the real detector intensities plotted in (**b**).

The conversion process from the supersaturated carbidic precursor state toward graphene is accompanied by another interesting effect, namely the decrease in intensity of certain ring-like diffraction features, as will be discussed in more detail in section 5. These features can neither be assigned to the discrete diffraction spots of the Ni_2_C |4772| superstructure nor to the integral order spots of the Ni(111) substrate. The corresponding diffraction intensities are highlighted in Fig. 7, along with the respective “powder diffractogram” obtained by direct azimuthal integration of the raw detector intensity data in the marked region (red transparent bar). This region was chosen in view of a minimized contribution of discrete point reflections of the Ni_2_C |4772| superstructure relative to the ring-like diffraction intensities. We note that their intensity contribution cannot be masked to 100% using this approach. By comparing the d-spacings of the rings to those of the Ni_2_C spots within the area, especially the intensities below ~2.0 Å are affected by the Ni_2_C point reflections within the bar. We further note that the optically enhanced b/w contrast in panel (a) provides no direct measure of the real detector intensities plotted in (b).

**Figure 8:**
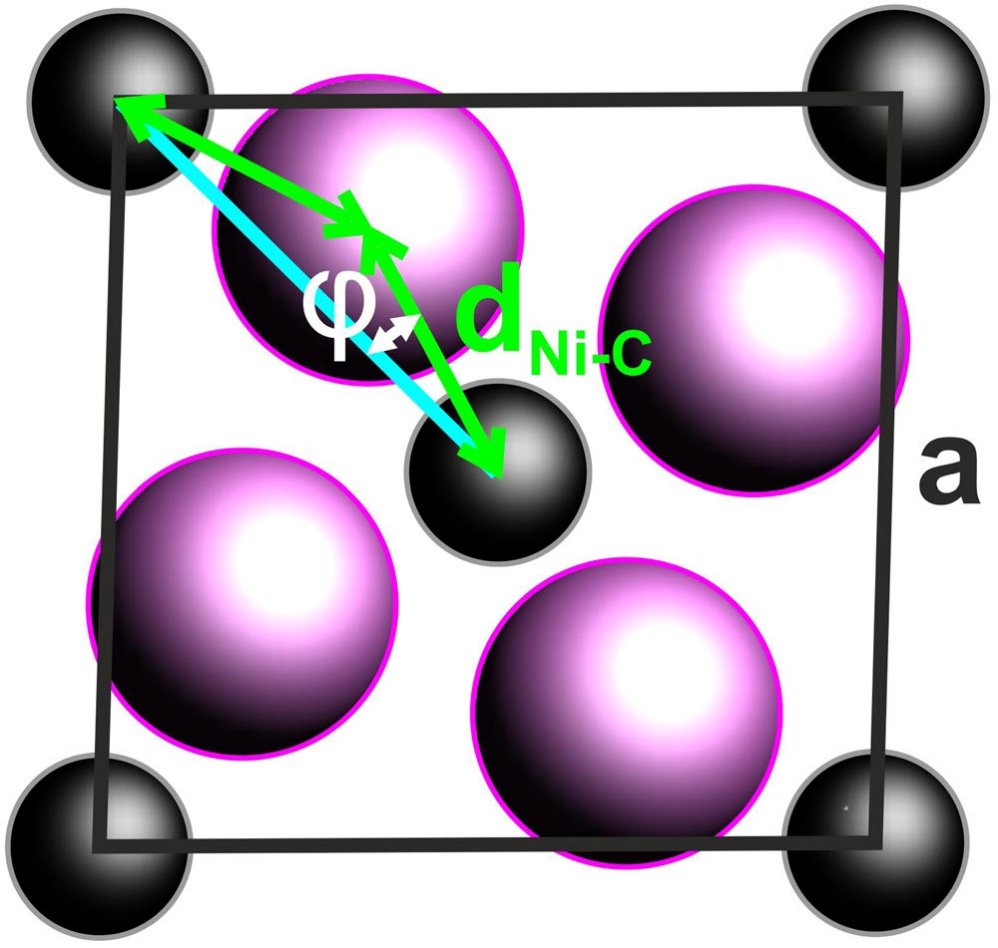
Dominant structural motif of the surface mesh and of the projected Ni_2_C bulk unit cell. In the case of a square planar structure and equal Ni-C bond lengths, the unit cell length a is related to the rotation angle φ as $a=2\sqrt{2}{d}_{{Ni}-C}{\rm{\cos }}\phi$. The fact that not all Ni-C bond lengths are exactly equal and a small triclinic distortion will lead to deviations from this purely geometrical consideration.

Based on these experimental results, we will now postulate a possible crystal structure for this hitherto unobserved and apparently metastable nickel-carbide phase. Since the experimental data are rather limited, we will have to confine our discussion to certain features that should exist in the crystal structure, rather than completely solving it. In analogy to surface oxide formation, i.e. a single 2D oxide monolayer forming during metal oxidation under certain non-ambient conditions, it seems reasonable to view the Ni_2_C surface carbide as the initial stage to bulk carbide formation. However, the related intensities appear to be partly developed already after the lowest exposure of 7.5 L, as will be shown in section 5, which might unfavour such a 3D nucleation of bulk carbide phases scenario. In any case, sufficiently flat 3D or 2D surface-covering structures with a certain abundance must be responsible for the ring-like features. The most prominent candidate structure would be Ni_3_C, because this is the only stable carbide found in the phase diagram. However, the observed d-spacings do not fit to the hexagonal Ni_3_C phase^31^. In addition, from the conditions under which we observe the new phase (supersaturated by ethylene exposure) one would expect that the carbon content should be higher than in Ni_2_C, which would rule out Ni_3_C from a compositional point of view. A comprehensive theoretical study on metastable nickel-carbides includes a phase stability investigation^32^, which shows that compounds containing more carbon than Ni_2_C are drastically less stable. These considerations, together with the experimental fact that the surface carbide’s composition is Ni_2_C, leads to the assumption that the presently discussed carbide also has a composition close to that. The pronounced structural motif of the surface carbide, the mesh, is assumed to also be present in such a bulk Ni_2_C phase. Gibson *et al*.^32^ have also investigated many different prototype structures for different metastable bulk phases in the Ni-C system by DFT. For Ni_2_C, the lowest energy structures are found for a rutile (tetragonal) and an orthorhombic (Fe_2_C-like) type. The projection along the shortest axis of both these structures shows an atomic arrangement which resembles the structural motif of the mesh here, see Fig. 8. The diffraction geometry used to obtain maps such as in Fig. 7, give access to reflections of type *hk0*, with the coordinate system used by us.

For the structures discussed here, the strongest reflection hk0 can be used to tentatively index the observed reflections, as shown in Table 1.

**Table 1:** Observed d-spacings and tentative indices, based on a Ni_2_C bulk structure as discussed in the main text.

d_obs_ (Å)	hkl
2.42	200
2.20	020
1.60	220

These reflections can be used to calculate the in-plane lattice parameters of the structure as a = 4.8(1) Å, b = 4.4(1) Å and γ = 92(3)°. Although the angle γ is nearly identical, the side lengths are considerably smaller than the average size of the mesh in the |4772| cell, which are a = 5.08 Å and b = 4.85 Å. This difference in cell parameters can easily be understood by considering that in the proposed bulk structures the Ni and C atoms are not in the same plane; by displacing the C atoms along the c-axis out of the ground plane and keeping the Ni-C bond length constant, the a and b axes will become smaller. Even in the case of a nearly planar 2D structure, such as the surface carbide, the mesh cell parameters can easily be adjusted through rotation of the 4 Ni atoms around the central carbon. This structural geometry feature allows accommodating strain, without changing the Ni-C bond lengths too much, over a relatively large range. The side lengths of an ideal square mesh scale with ~cos(φ) and by changing it from 20 to about 30 degrees, the side lengths change by about 10%. This strain-relieving mechanism plays an important role in the structure formation of the surface carbide and allows to build a nearly strain-free large coincidence cell.

As mentioned above, the maps obtained by us only give a 2D, projected view of the crystal structure. Details of the structure along the surface normal are contained in Bragg reflections which have not been observed by us, possibly because of the high scattering angles at which these appear. Most likely the structure is heavily textured having its preferred orientation along the substrate’s surface normal, which complicates the detection of out-of-plane diffraction spots even more.

As SXRD does not allow for identifying the corresponding real-space structures unambiguously on a tentative model basis, it will be necessary to deliberately look with STM for minority structures within the long-range ordered 2D Ni_2_C surface carbide structure, especially under the highly C-supersaturated conditions used in this work. Possible hints for the coexistence of quasi 3D or bulk-like carbide species and the single-layer Ni_2_C surface carbide can also be extracted from Ni(111)-based XPS literature data showing an additional C1s component at ≤283.0 eV binding energy, well below the value of 2D Ni_2_C surface carbide (~283.3 eV)^33^. The assignment of such low BE components to metastable bulk carbidic phases has been made in Lit.^34^.

### Overview of surface structure changes with increasing carbon saturation and final graphene formation

**Figure 9:**
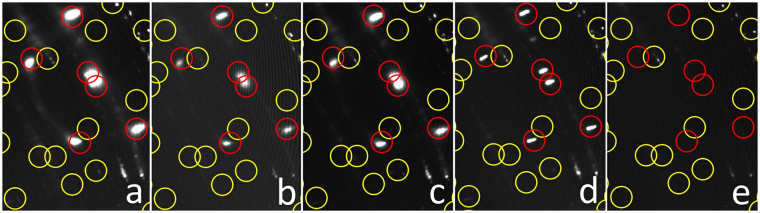
Representation of the “spot splitting region” in the hk maps of the full experimental sequence with increasing ethylene exposition and final decomposition of the supersaturated carbidic precursor toward graphene. (**a**) 7.5 L ethylene deposited on the quasi-clean Ni(111) crystal at 573 K, resulting in a Ni_2_C superstructure with 0.45 ML carbon coverage; (**b**) 379 L ethylene at 673 K; (**c**) 1353 L ethylene at 723 K; (**d**) 6316 L ethylene at 773/673 K; (**e**) thermal annealing in vacuum at 703 K after 2500 sec. Red circles: main |4772| coincidence cell reflections and its 6 symmetry related domains, along with the |4772|-specific peak splitting. Yellow circles: expected additional weak spots due to the large |4772| coincidence cell.

**Figure 10:**
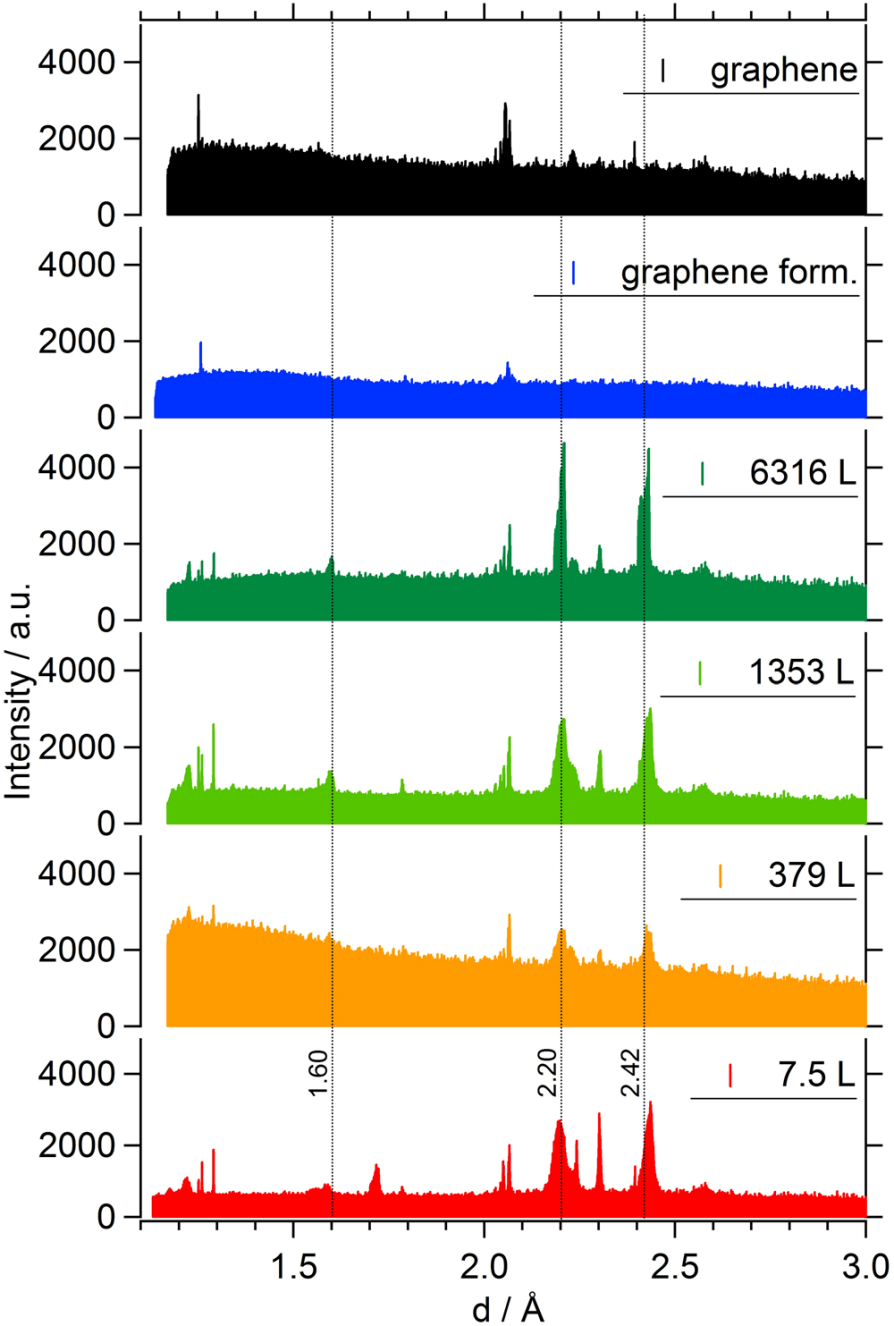
Development of the ring-like diffraction features as derived from the hk maps of the full experimental sequence with increasing ethylene exposition and final decomposition of the supersaturated carbidic precursor toward graphene, plotted as “powder diffraction data” versus the respective d-spacing value. Red: 7.5 L ethylene deposited on the quasi-clean Ni(111) crystal at 573 K; yellow: 379 L ethylene at 673 K; bright green: 1353 L ethylene at 723 K; dark green: 6316 L ethylene at 773/673 K; blue: during thermal annealing in vacuum at 703 K after 2500 sec; black: after cooling of the fully graphene covered sample to room temperature.

Eventually, Figs 9 and 10 provide a more detailed picture of the structural developments upon increasing carbon supersaturation of the surface and the finally resulting graphene nucleation and growth process, based on the respective intensity changes in the hk-maps.

The most prominent effect in Fig. 9 is the gradually visible peak splitting of the (6, 4) carbide spots of the final |4772| superstructure with increasing exposure and temperature. This suggests a dynamic reordering process of the Ni_2_C top layer, which is, around 673 K, just at the edge of decay and thermodynamically only stabilized due to the permanent presence of the 2 × 10^−6^ mbar ethylene gas phase. In order to explain the increasing long-range ordering, a continuous structural reformation process is suggested, which is necessarily accompanied by ongoing slow carbon loss to subsurface regions, further C supersaturation of near-surface bulk regions and simultaneous structural “ripening” of coherent |4772| domains within the carbide top layer. Also, the nucleation of bulk-like Ni_2_C domains, giving rise to the diffraction-ring-like features discussed in results and discussion section 4., can be rationalised on this basis. Upon thermal annealing of the supersaturated carbidic precursor in vacuo at 703 K, quantitative loss of all |4772| related point reflections is observed (Fig. 9 panel e and Fig. 6), indicating complete structural transition to the 2 ML carbon, i.e. fully graphene-covered, final state described in detail in results and discussion section 3.

Finally, Fig. 10 shows a plot of the integrated raw detector intensities within the marked sector of Fig. 7 (in red) for the full set of preparations and the subsequent thermal annealing step at 703 K. The transition from the metastable carbidic precursor state to the graphene-covered surface is also accompanied by the disappearance of the diffraction intensities corresponding to d = 2.42, 2.20 and 1.60 Å (dashed lines, dark green to black diffractogram in Fig. 9). The reflections below 1.3 Å were not considered, since they rather appear to stem from co-integrated point reflections of the |4772| superstructure, which vanishes simultaneously upon thermal annealing, as described above (Fig. 6 and 9).

The goal was therefore to highlight the “ring-specific” intensities and their behaviour upon decomposition of the precursor with a minimized contribution of crystal and Ni_2_C surface truncation rod intensities. Clearly, a strong intensity loss of the marked diffraction peaks (dashed lines) is observed upon isothermal annealing of the supersaturated carbidic precursor in vacuo at 703 K (Fig. 10, from dark green to blue panel). Interestingly, upon cooling from 703 K down to room temperature, some of the “carbidic” intensities seem to be re-established, despite the fact that the state of the blue diffractogram corresponds to an already fully graphene-covered surface. This might be caused by re-formation of carbidic domains underneath the graphene layer, leading to layered carbide-graphene structures similar to those described in^15^. Apparently, the species causing the ring-like diffraction intensities are equally contributing to the graphene top layer nucleation and growth, as compared to the ordered/epitaxial Ni_2_C domains.

## Conclusions

A fully surface-covering epitaxial graphene overlayer was formed by thermal decomposition of a carbon-supersaturated/Ni_2_C surface carbide-covered Ni(111) precursor state in absence of external carbon supply from the gas phase.

Both the precursor state, the transition process and the resulting fully graphene-covered Ni(111) surface were characterized by surface X-ray diffraction (SXRD). The most prominent, fingerprint-like, signature for the different surface structures is seen in the corresponding specular rod. The X-ray data allow to unambiguously identify the |4772| coincidence cell as the correct one for the Ni_2_C surface carbide. DFT calculations of the |4772| structure are in perfect agreement with the SXRD data and show essentially a clock-reconstructed network whereby the carbon atoms are slightly closer to the underlying substrate, of which the last layer of Ni atoms show minor in-plane relaxations.

The perfectly epitaxial graphene layer nucleates and grows via a combined C-antisegregation/ Ni_2_C surface carbide decomposition process without external C supply at 703 K. As described in earlier work^7^, this low temperature growth mode does not lead to a mixture of epitaxial and rotated (non-epitaxial) graphene domains. The CTR data indicate the simultaneous presence of 2 high-symmetry configurations of graphene domains, namely top-fcc and bridge-top. The strong modulation of the (0, 0) specular rod nevertheless allows for a clear assignment of the resulting 2 ML graphene layer.

## Electronic supplementary material

Supplementary Information
