# Supplementary material for: Role of Precursor Carbides for Graphene Growth on Ni(111)
Source: Sci Rep. 2018 Feb 8;8:2662. doi: 10.1038/s41598-018-20777-4 (PMC5805774; doi:10.1038/s41598-018-20777-4)
Supplement: Supplementary file 1 — Supplementary Information [file 41598_2018_20777_MOESM1_ESM.pdf]

Supplementary Information

Role of Precursor Carbides for Graphene Growth on Ni(111)

Raffael Rameshan[1,2], Vedran Vonk[3], Dirk Franz[4], Jakub Drnec[5], Simon Penner[1], Andreas Garhofer [6], Florian Mittendorfer[6], Andreas Stierle[3], Bernhard Klötzer[1*]

[1]*Institute of Physical Chemistry, University of Innsbruck, Innrain 80-82, A-6020 Innsbruck, Austria*

[2]*Department of Inorganic Chemistry, Fritz-Haber-Institute of the Max-Planck-Society, Faradayweg 4–6, D-14195 Berlin, Germany*

[3] *Deutsches Elektronen-Synchrotron (DESY), D-22607 Hamburg, Germany*

[4] *Fachbereich Physik, Universität Hamburg, D-22607 Hamburg, Germany*

[5]*ESRF-The European Synchrotron, Avenue des Martyrs 71, 38000 Grenoble, France*

[6] *Institut für Angewandte Physik, Center for Computational Materials Science, Technische Universität Wien, Wiedner Hauptstrasse 8-10, A-1040 Wien*

In this supplementary information section, the atomic co-ordinates of the different crystallographic structures are listed. Given are the parameters obtained for the quasi-clean Ni(111) surface in Table S1, the results of the Ni_2C |4772| coincidence structure on Ni(111) in Table S2, and the graphene-covered Ni(111) structure in Table S3.

Table S1. Fractional co-ordinates of the refined quasi-clean Ni(111) surface. The cell parameters are: $a = b = 0.2489$ nm, $c = 0.61037$ nm, $\alpha = \beta = 90°, \gamma = 120°$ and the last layer of atoms in the bulk lies at z=0. The Debye-Waller parameters B are given in units of Å^2. The occupancy, θ, is slightly less than 1, as explained in the main text. The best fit gave R = 0.118.

Atom	x	y	z	$B\parallel$	$B\perp$	θ
Ni	0	0	0.336	0.33	0.33	0.95

Table S2. Fractional co-ordinates of the refined Ni_2C |4772| coincidence cell. The cell parameters are: $a = 1.51571$ nm, $b = 1.55615$ nm, $c = 0.61037$ nm, $\alpha = \beta = 90°, \gamma = 69.2°$. The Ni atoms at z=0.00017 form the terminating and laterally undistorted substrate layer and the last layer of atoms in the bulk lies at z=-0.333. The Ni layer above that, around z=0.33, contains lateral relaxations, as explained in the main text. The Debye-Waller parameters B are given in units of Å^2. The best fit gave R = 0.131.

Atom	x	y	z	$B\|$	$B\perp$
Ni	0.87506	0.05929	0.69625	3.07	0.49
Ni	0.54207	0.05828	0.69226	3.07	0.49
Ni	0.21441	0.06196	0.67235	3.07	0.49
Ni	0.69554	0.10589	0.67047	3.07	0.49
Ni	0.36045	0.11258	0.67790	3.07	0.49
Ni	0.02823	0.10916	0.68196	3.07	0.49
Ni	0.01915	0.27343	0.69483	3.07	0.49
Ni	0.68291	0.27273	0.67928	3.07	0.49
Ni	0.35491	0.27785	0.69493	3.07	0.49
Ni	0.86789	0.22396	0.68154	3.07	0.49
Ni	0.53601	0.22396	0.67957	3.07	0.49
Ni	0.19868	0.22796	0.67330	3.07	0.49
Ni	0.76265	0.39084	0.69698	3.07	0.49
Ni	0.43254	0.39336	0.68238	3.07	0.49
Ni	0.10064	0.39577	0.68214	3.07	0.49
Ni	0.91797	0.44097	0.67828	3.07	0.49
Ni	0.58250	0.44012	0.67482	3.07	0.49
Ni	0.24995	0.44586	0.67310	3.07	0.49
Ni	0.90674	0.60588	0.68795	3.07	0.49
Ni	0.57409	0.60764	0.68279	3.07	0.49
Ni	0.24417	0.61104	0.69662	3.07	0.49
Ni	0.75677	0.55591	0.67754	3.07	0.49
Ni	0.42484	0.56051	0.67792	3.07	0.49
Ni	0.08823	0.56069	0.67385	3.07	0.49
Ni	0.98807	0.72833	0.69129	3.07	0.49
Ni	0.65193	0.72357	0.69479	3.07	0.49
Ni	0.32367	0.72807	0.67677	3.07	0.49
Ni	0.80801	0.77344	0.67547	3.07	0.49
Ni	0.47104	0.77636	0.67837	3.07	0.49
Ni	0.13941	0.77776	0.68058	3.07	0.49
Ni	0.79334	0.93895	0.68030	3.07	0.49
Ni	0.46449	0.94244	0.69381	3.07	0.49
Ni	0.13253	0.94233	0.69644	3.07	0.49
Ni	0.97868	0.89235	0.67975	3.07	0.49
Ni	0.64630	0.88873	0.67875	3.07	0.49
Ni	0.31201	0.89549	0.67008	3.07	0.49
C	0.11421	0.16589	0.60140	4.00	1.98
C	0.44876	0.16794	0.61240	4.00	1.98
C	0.33942	0.50211	0.60868	4.00	1.98
C	0.22750	0.83359	0.60291	4.00	1.98
C	0.89210	0.83533	0.60131	4.00	1.98
C	0.78007	0.16811	0.60461	4.00	1.98
C	0.66762	0.50001	0.60878	4.00	1.98
C	0.55750	0.83377	0.61218	4.00	1.98
C	0.00265	0.50058	0.59948	4.00	1.98
C	0.00377	0.00000	0.70070	4.00	1.98
C	0.34223	0.00276	0.61743	4.00	1.98
C	0.22834	0.33517	0.66390	4.00	1.98
C	0.11607	0.66838	0.68906	4.00	1.98
C	0.77909	0.66596	0.67242	4.00	1.98
C	0.66622	0.99813	0.63175	4.00	1.98
C	0.55682	0.33197	0.61046	4.00	1.98

C	0.89105	0.33318	0.69369	4.00	1.98
C	0.44979	0.66787	0.60818	4.00	1.98
Ni	0.02204	0.41472	0.33515	4.00	0.12
Ni	0.07614	0.24346	0.33272	4.00	0.12
Ni	0.12103	0.07389	0.33801	4.00	0.12
Ni	0.00000	1.00000	0.36978	4.00	0.12
Ni	0.05003	0.82804	0.32668	4.00	0.12
Ni	0.10024	0.65919	0.36056	4.00	0.12
Ni	0.14844	0.48598	0.32789	4.00	0.12
Ni	0.19881	0.31793	0.34701	4.00	0.12
Ni	0.24603	0.14537	0.33047	4.00	0.12
Ni	0.17268	0.90115	0.33078	4.00	0.12
Ni	0.22156	0.73101	0.33633	4.00	0.12
Ni	0.27042	0.55802	0.33353	4.00	0.12
Ni	0.32054	0.38724	0.33106	4.00	0.12
Ni	0.36883	0.21551	0.33729	4.00	0.12
Ni	0.41643	0.04411	0.33636	4.00	0.12
Ni	0.29418	0.97647	0.33062	4.00	0.12
Ni	0.34238	0.80534	0.33330	4.00	0.12
Ni	0.39156	0.63577	0.33229	4.00	0.12
Ni	0.43865	0.46614	0.33802	4.00	0.12
Ni	0.48866	0.29575	0.33533	4.00	0.12
Ni	0.53583	0.12510	0.33917	4.00	0.12
Ni	0.46620	0.87434	0.34100	4.00	0.12
Ni	0.51310	0.70385	0.33437	4.00	0.12
Ni	0.56307	0.53423	0.33657	4.00	0.12
Ni	0.61045	0.36364	0.33180	4.00	0.12
Ni	0.65883	0.19471	0.33198	4.00	0.12
Ni	0.70672	0.02261	0.33514	4.00	0.12
Ni	0.58472	0.95437	0.33419	4.00	0.12
Ni	0.63329	0.78372	0.33559	4.00	0.12
Ni	0.68124	0.61136	0.33261	4.00	0.12
Ni	0.73101	0.44101	0.33259	4.00	0.12
Ni	0.78018	0.26747	0.33868	4.00	0.12
Ni	0.82770	0.09789	0.33104	4.00	0.12
Ni	0.75534	0.85425	0.33010	4.00	0.12
Ni	0.80268	0.68142	0.35170	4.00	0.12
Ni	0.85288	0.51285	0.32770	4.00	0.12
Ni	0.90100	0.34048	0.36438	4.00	0.12
Ni	0.95112	0.17063	0.32647	4.00	0.12
Ni	0.88007	0.92514	0.33862	4.00	0.12
Ni	0.92421	0.75565	0.33295	4.00	0.12
Ni	0.97847	0.58441	0.33467	4.00	0.12
Ni	0.02439	0.08130	0.00017	0.30	0.30
Ni	0.00000	0.66667	0.00017	0.30	0.30
Ni	0.04878	0.49593	0.00017	0.30	0.30
Ni	0.09756	0.32520	0.00017	0.30	0.30
Ni	0.14634	0.15447	0.00017	0.30	0.30
Ni	0.07317	0.91057	0.00017	0.30	0.30
Ni	0.12195	0.73984	0.00017	0.30	0.30
Ni	0.17073	0.56911	0.00017	0.30	0.30
Ni	0.21951	0.39837	0.00017	0.30	0.30
Ni	0.26829	0.22764	0.00017	0.30	0.30
Ni	0.31707	0.05691	0.00017	0.30	0.30
Ni	0.19512	0.98374	0.00017	0.30	0.30
Ni	0.24390	0.81301	0.00017	0.30	0.30
Ni	0.29268	0.64228	0.00017	0.30	0.30
Ni	0.34146	0.47154	0.00017	0.30	0.30
Ni	0.39024	0.30081	0.00017	0.30	0.30
Ni	0.43902	0.13008	0.00017	0.30	0.30
Ni	0.36585	0.88618	0.00017	0.30	0.30
Ni	0.41463	0.71545	0.00017	0.30	0.30

Ni	0.46341	0.54472	0.00017	0.30	0.30
Ni	0.51220	0.37398	0.00017	0.30	0.30
Ni	0.56098	0.20325	0.00017	0.30	0.30
Ni	0.60976	0.03252	0.00017	0.30	0.30
Ni	0.48780	0.95935	0.00017	0.30	0.30
Ni	0.53659	0.78862	0.00017	0.30	0.30
Ni	0.58537	0.61789	0.00017	0.30	0.30
Ni	0.63415	0.44715	0.00017	0.30	0.30
Ni	0.68293	0.27642	0.00017	0.30	0.30
Ni	0.73171	0.10569	0.00017	0.30	0.30
Ni	0.65854	0.86179	0.00017	0.30	0.30
Ni	0.70732	0.69106	0.00017	0.30	0.30
Ni	0.75610	0.52033	0.00017	0.30	0.30
Ni	0.80488	0.34959	0.00017	0.30	0.30
Ni	0.85366	0.17886	0.00017	0.30	0.30
Ni	0.90244	0.00813	0.00017	0.30	0.30
Ni	0.78049	0.93496	0.00017	0.30	0.30
Ni	0.82927	0.76423	0.00017	0.30	0.30
Ni	0.87805	0.59350	0.00017	0.30	0.30
Ni	0.92683	0.42276	0.00017	0.30	0.30
Ni	0.97561	0.25203	0.00017	0.30	0.30
Ni	0.95122	0.83740	0.00017	0.30	0.30

Table S3. Fractional co-ordinates of the refined graphene-covered Ni(111) surface. The cell parameters are: $a = b = 0.2489$ nm, $c = 0.61037$ nm, $\alpha = \beta = 90°, \gamma = 120°$ and the last layer of atoms in the bulk lies at z=0. The Debye-Waller parameters B are given in units of Å^2 and the occupancy, θ, as a dimensionless value. The best fit gave R = 0.169. Carbon atoms of the bridge-top domain are labeled 1, those with the top-fcc geometry are labeled 2.

Atom	x	y	z	$B\parallel$	$B\perp$	θ
C1	0.333	0.1667	0.67598	10	2.3	0.5
C1	0.667	0.8333	0.67598	10	2.3	0.5
C2	0.667	0.333	0.67598	10	2.3	0.5
C2	0.000	0.000	0.67958	10	2.3	0.5
Ni	0.000	0.000	0.33355	0.7	2.0	0.96
